# DESP: Deep Enhanced Sampling of Proteins’ Conformation Spaces Using AI-Inspired Biasing Forces

**DOI:** 10.3389/fmolb.2021.587151

**Published:** 2021-05-04

**Authors:** Emmanuel Oluwatobi Salawu

**Affiliations:** Machine Learning Solutions Lab, Amazon Web Services (AWS), Herndon, VA, United States

**Keywords:** conformation space, deep neural network, protein, molecular dynamics simulation, variational autoencoder

## Abstract

The molecular structures (i.e., conformation spaces, CS) of bio-macromolecules and the dynamics that molecules exhibit are crucial to the understanding of the basis of many diseases and in the continuous attempts to retarget known drugs/medications, improve the efficacy of existing drugs, or develop novel drugs. These make a better understanding and the exploration of the CS of molecules a research hotspot. While it is generally easy to computationally explore the CS of small molecules (such as peptides and ligands), the exploration of the CS of a larger biomolecule beyond the local energy well and beyond the initial equilibrium structure of the molecule is generally nontrivial and can often be computationally prohibitive for molecules of considerable size. Therefore, research efforts in this area focus on the development of ways that systematically favor the sampling of new conformations while penalizing the resampling of previously sampled conformations. In this work, we present *Deep Enhanced Sampling of Proteins’ Conformation Spaces Using AI-Inspired Biasing Forces* (DESP), a technique for enhanced sampling that combines molecular dynamics (MD) simulations and deep neural networks (DNNs), in which biasing potentials for guiding the MD simulations are derived from the KL divergence between the DNN-learned latent space vectors of [a] the most recently sampled conformation and those of [b] the previously sampled conformations. Overall, DESP efficiently samples wide CS and outperforms conventional MD simulations as well as accelerated MD simulations. We acknowledge that this is an actively evolving research area, and we continue to further develop the techniques presented here and their derivatives tailored at achieving DNN-enhanced steered MD simulations and DNN-enhanced targeted MD simulations.

## Introduction

The functions of biomolecules are encoded in their structures and dynamics (Council and others 1989; Karplus and Kuriyan, 2005; Yang et al., 2014). And there are innumerable pieces of evidence linking the basis of many diseases to anomalies in the structures and the dynamics of the molecules that are involved in the biological systems that the diseases affect (McCafferty and Sergeev, 2016; Chiti and Dobson, 2017; Guo et al., 2017; Hartl, 2017; Tramutola et al., 2017; Salawu, 2018a; Ittisoponpisan et al., 2019; Laskowski et al., 2020) because the normal functioning of the biological systems depends on the molecules’ proper structures and dynamics. Furthermore, the various structures that a molecule can take (i.e., the molecule’s conformation space, CS) and their associated MD are not only of vital importance in deciphering of many diseases (Salawu, 2018a; Salawu, 2018b) but are also crucial in the drug development efforts targeted at curing or managing many diseases (Carlson and McCammon, 2000; Lee et al., 2018; Pawełand and Caflisch, 2018; Wang et al., 2018; Lin et al., 2020). These recognitions have motivated extensive efforts in the field of structural biochemistry and form the rationale for many structural biology studies (such as through X-ray crystallography, NMR, and Cryo-EM) and the creation of the Protein Data Bank (Berman et al., 2000) as well as other databases for molecular structures. Nonetheless, considerable challenges exist because the solely static molecular structures obtained through the wet laboratory approaches alone (such as the ones listed above) often fall short of providing enough insights into the dynamics of the molecules of interest. These challenges have led to the growing roles and the increasing importance of computational approaches, such as molecular dynamics (MD) simulations, that are often used for studying the dynamic behaviors of molecules and their interactions with other molecules as well as for exploring much wider CS of the molecules of interest.

While it is generally easy to computationally explore the CS of small molecules (such as peptides and ligands), the exploration of the CS of larger a biomolecule beyond the local energy well and beyond the initial equilibrium structure of the molecule is generally nontrivial (Shaw et al., 2008; Shaw et al., 2009) and can often be computationally prohibitive for a molecule of considerable size. These difficulties arise from the existence of energy barriers between different states that the molecule could assume, thereby hindering the movement of the molecule from one structural state to another (Hamelberg et al., 2004; Hénin and Chipot, 2004; Salawu, 2020). At this point, it is important to acknowledge existing efforts targeted at removing, avoiding/sidestepping, lowering, or surmounting these energy barriers, thereby achieving enhanced sampling of the CS of molecules. Therefore, we recognize some of the previous publications in this domain and highlight them in the next paragraphs.

Most of the existing popular approaches for achieving enhanced sampling may be broadly viewed in two categories, namely: those that require the user to specify well-defined collective variables (CVs)/reaction coordinates (RCs) (Babin et al., 2008; Laio and Gervasio, 2008; Bussi and Laio, 2020) and those that do not require the user to explicitly specify the CV/RC (Sugita and Okamoto, 1999; Hamelberg et al., 2004; Moritsugu et al., 2012; Harada and Kitao, 2013; Miao et al., 2015; Chen and Ferguson, 2018; Salawu, 2020). Reconnaissance meta-dynamics uses a self-learning algorithm for accelerated dynamics and is capable of handling a large number of collective variables by making use of bias potentials created as a function of individual locally valid CVs that are then patched together to obtain the sampling across a large number of collective variables (Tribello et al., 2010). Some of the challenges of reconnaissance meta-dynamics such as those associated with the creation of bias potentials as a function of individual locally valid CVs could be addressed by any technique that could potentially learn a compressed representation of those CVs and efficiently explore the combined CVs together in the compressed space. This is the subject of an actively growing research area that leverages the powers of deep neural networks (DNNs)/machine learning (ML). Bridging the fields of enhanced sampling and ML, Bonati et al. (2019) developed DNN-based variationally enhanced sampling that uses neural networks to represent the bias potential in a variational learning scheme that makes it possible for the efficient exploration of even high-dimensional free energy surfaces. In a similar way, reweighted autoencoded variational Bayes (RAVE) models MD simulation trajectories using the VAE whereby the learned distribution of the latent space variable is used to add biasing potentials, thereby penalizing the repeated sampling of the most favorable frequently visited states (Ribeiro et al., 2018). Although other enhanced sampling methods implement the biasing protocol in two steps, RAVE’s identification of the RC and its derivation of unbiased probability distribution occur simultaneously. And through the systematic use of the Kullback–Leibler (KL) divergence metric, RAVE can identify physically meaningful RCs from among a group of RCs explored.

In addition to the efforts mentioned above, the combination of well-tempered meta-dynamics and time-lagged independent component analysis to study rare events and explore complex free energy landscapes have also been looked into (McCarty and Parrinello, 2017). Since the initial choice of CVs for meta-dynamics is often suboptimal, the work shows the finding of new and optimal CVs with better convergence properties by the analysis of the initial trajectory using time-lagged independent component analysis (McCarty and Parrinello, 2017). However, a more recent study has shown that rather than using linear dimension reduction methods (such as independent component analysis) a modified autoencoder could more accurately encode the low dynamics of the underlying stochastic processes of MD simulations better than linear dimension reduction methods (Wehmeyer and Noé, 2018). Indeed, there are continuous and growing efforts in the combinations of DNN models and MD simulations in the enhancement of the sampling of molecules’ CS and other various aspects of molecular sciences (Allison, 2020; Salawu, 2020; Sidky et al., 2020).

In this work, we present *Deep Enhanced Sampling of Proteins’ Conformation Spaces Using AI-Inspired Biasing Forces* (DESP), which also combines DNNs and MD simulations to create a robust technique for enhanced sampling of CS of molecules. Here, a DNN model is trained alongside MD simulations of the molecule of interest such that the models learn a compressed representation of the sampled structures of the molecule. The latent space vectors of the DNN model are then used in ways that provide useful information for inferring appropriate biasing potentials that are then used for guiding the MD simulations, thereby allowing efficient sampling of the molecule’s CS. More specifically, the use of the KL divergence between the VAE’s latent vectors of the current conformation (obtained from the MD simulations) and the VAE’s latent vectors of the known, previously sampled, conformations makes it possible to bias the MD simulation away from visiting previously sampled conformations and rather toward visiting previously unsampled conformations.

The AI-based enhanced sampling approach presented in this work is not dependent on having prior knowledge of the molecule’s CS distribution and does not require any careful selection of collective variables. Therefore, this approach is very promising, given that the selection of appropriate collective variables is often very challenging (Tribello et al., 2010), and there is no well-defined solution that can fit all situations/all molecular systems. Rather than requiring manual specification of the collective variables to use, DESP, by itself, learns the compressed representation of the molecular system of interest and derives biasing potentials based on the distribution of the molecule’s conformations in that compressed representation space. The results obtained show that DESP outperforms both conventional and accelerated MD simulations, and efficiently samples wider CS than conventional and accelerated MD simulations. Furthermore, the ideas in DESP are generalizable and may be used for implementing other forms of biased MD simulations including targeted and steered MD simulations. In the next section, we present the methods that make DESP possible and thereafter the overall DESP algorithm.

## Materials and Methods

### Protein Molecules Used

We began with a smaller protein/peptide (alanine dodecapeptide with 12 alanine residues, A_12_) and modeled its 3D structure using RPBS (Alland et al., 2005). The small size of alanine dodecapeptide helped in the initial testing and fine-tuning of DESP. In addition to A_12_, we obtained a solution nuclear magnetic resonance (NMR) structure of GB98 that was expressed in *Escherichia coli* BL21 (DE3) from the Protein Data Bank (Berman et al., 2000), PDB ID: 2lhd (He et al., 2012). GB98 was selected because of its relatively small/medium size and because of the presence of the various secondary structure types (namely, alpha-helix, beta-sheet, and coils) in it. On the other hand, any protein could be used for the demonstration of the functionality of DESP, and the ones used here are just examples.

### Creation of the Initial Molecular Systems

Assignment of appropriate residues' charges and protonation states were handled using PDB2PQR (Dolinsky et al., 2007; Jurrus et al., 2018). Using AmberTools18's tLeap (Pearlman et al., 1995; Case et al., 2005; Salomon-Ferrer et al., 2013a; Salomon-Ferrer et al., 2013b), ff14SB (Maier et al., 2015) force-fields for the proteins, and ions234lm_126_tip3*p* for the ions and the water molecules (Li and Merz, 2014), we created explicitly solvated molecular systems for A_12_’s and GB98’s molecular dynamics (MD) simulations with OpenMM (Eastman et al., 2017) containing 2068 TIP3P water molecules (42.38Å × 48.80Å × 47.15Å box size, for A_12_) or 9,981 (101.10Å × 94.35Å × 98.34Å box size, for GB98) TIP3P water molecules.

### Energy Minimization and Heating

Each of the molecular systems was energy-minimized using OpenMM (Eastman et al., 2017). The energy minimizations were done in two stages—weakly (2.5 kcal/mol/Å^2^) restraining all the alpha carbon atoms in the first stage, and without any restraints in the second stage. With the weak restraints (2.5 kcal/mol/Å^2^) reapplied on the alpha carbon atoms, the molecular systems were steadily heated to a temperature of 310 K in a canonical ensemble using the Langevin thermostat (Pastor et al., 1988).

### Conventional Molecular Dynamics Simulations

During both the equilibration and production runs, we controlled the systems' temperatures and pressures using the Langevin thermostat (Pastor et al., 1988) with a collision frequency of 2ps^−1^ and the Monte Carlo barostat (Chow and Ferguson, 1995; Åqvist et al., 2004), respectively. Full electrostatic interaction energies were calculated using the particle mesh Ewald method (Darden et al., 1993). A cutoff distance of 10Å and a cubic spline switch function were used when calculating nonbonded interactions. All bonds in which at least one atom is hydrogen are constrained using the SHAKE algorithm (Ryckaert et al., 1977). All production run MD simulations were performed at 2 femtoseconds time step. Overall, the results from 800 ns of conventional MD simulations, 800 ns of accelerated MD simulations, and 280 ns of DESP MD simulations are presented in this work for each of the A_12_ and the GB98 molecular systems.

### Representations of the Molecules for Deep Learning Modeling

**FIGURE 1:**
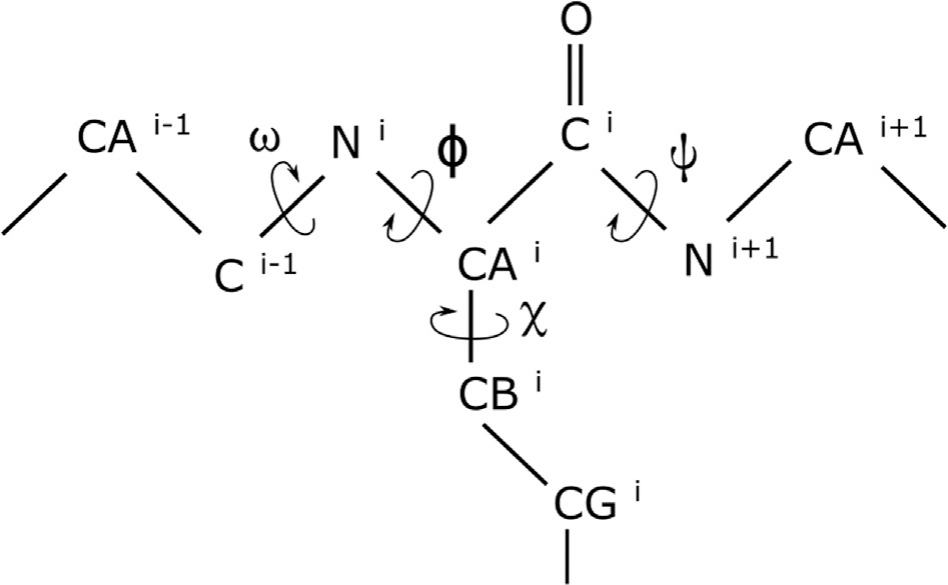
Dihedral angles in a short segment of a protein. While the omega (ω), phi (ϕ), and psi (ψ) dihedral angles are in the proteins backbone, the chi_1_ (χ1) dihedral angle is at the beginning of an amino acid’s side chain.

Since considerable changes in the conformation of biomolecules can be captured by variations in the dihedral angles of the molecules (Salvador, 2014; Cukier, 2015; Ostermeir and Zacharias, 2014; Lemke and Peter, 2019), we represent a molecule’s conformation by the cosine and the sine of the dihedral angles (Mu et al., 2005) of that conformation. For these, we make use of the omega (ω), phi (ϕ), psi (ψ), and chi_1_ (χ1) dihedral angles (with examples illustrated in Figure 1). Although using both the cosine and the sine of each of the dihedral angles doubles the dimensionality, it helps in removing the adverse effect that the periodicity of the dihedral angles would have had on the modeling. Extensive details of the benefits of using the dihedral angles (Lemke and Peter, 2019) and of simultaneously using both their cosines and sines have been documented elsewhere (Mu et al., 2005) and, for brevity, are not repeated here.

### DNN Architecture: Variational Autoencoder

**FIGURE 2:**
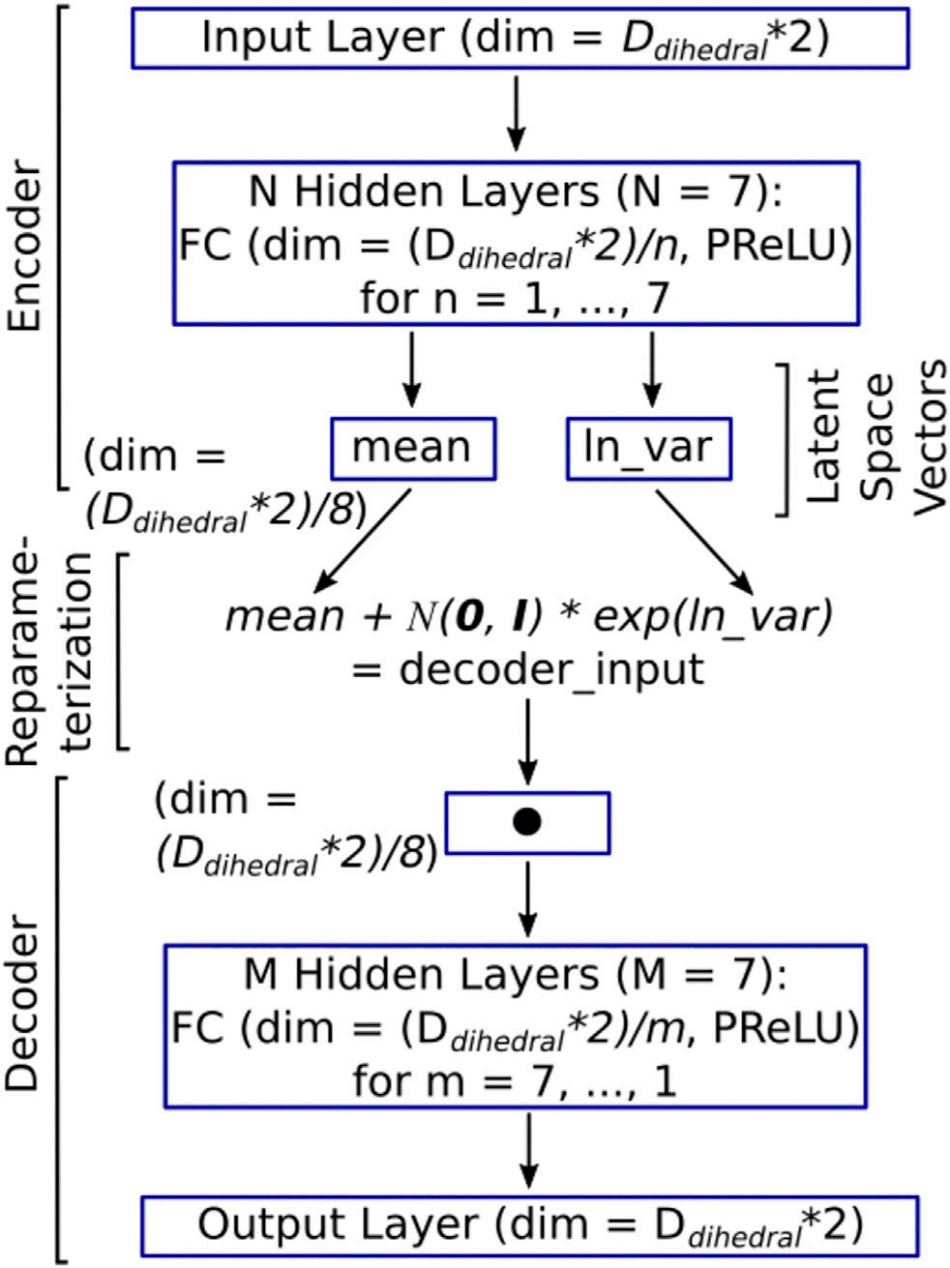
The architecture of the DESP’s VAE model. The dimension of the input layer (as well as the output layer) is two times the number of dihedral angles because both the cosine and the sine of each of the dihedral angles are used to deal with periodicity issues (Mu et al., 2005). Each of the hidden layers is a fully connected (FC) layer, followed by parameterized rectified linear units (PReLUs). The latent space between the encoder and the decoder has a dimension that is one-eighth of the input dimension to learn a compressed representation of the molecules in a reduced dimension. The encoder and the decoder are connected through the re-parametrization trick wherein samples are selected from the standard normal distribution, N(0, I), and then scaled by the variance and shifted by the mean.

Our DNN of the type variational autoencoder (VAE) has a simple architecture, as shown in Figure 2. The input layer takes both the cosine and the sine of the dihedral angles’ representation of the molecular conformation (giving rise to a vector of dimension D_Dihedrals_*2) as input. The input layer is followed by N hidden layers (where *N* = 7 in the current case). Each of the hidden layers, numbered *n* = [1, 2 … N], has D_Dihedrals_/n nodes. The next layer is made up of two latent space vectors, each of size (D_Dihedrals_*2)/(N + 1), which is (D_Dihedrals_*2)/8 in the current case. The first latent space vector represents the mean for the Gaussian distribution that the latent space encodes (i.e., mean in Figure 2), while the second vector represents the natural logarithm of the variance for the Gaussian distribution that the latent space encodes (i.e., ln_var in Figure 2). The DNN architecture up to this point is the encoder (Figure 2).

The decoder, which is like a mirror image of the encoder, begins with an input layer with (D_Dihedrals_*2)/(N + 1) nodes and is followed by M hidden layers (where M = 7 in the current case). Each of the hidden layers, numbered m = [M, M-1 … 1], has (D_Dihedrals_*2)/m nodes. The output (which is the final) layer emits the reconstructed cosine and sine of the dihedral angles of the molecular conformation that was passed in as input. To allow the passage of backpropagation signals through the entire VAE (i.e., from the decoder to the encoder), we connect the encoder and the decoder by a re-parameterization trick that is made up of an equation that takes the output of the encoder (namely, the vector of mean, and the vector of the logarithm of variance) as an input and uses it to sample from the corresponding normal distributions. This is done indirectly by initially drawing samples from the standard normal distribution. The samples drawn are then scaled and shifted accordingly using the variance vector and the mean vector, thereby obtaining the intended distribution (see the re-parameterization expression in Figure 2). The output of the re-parameterization is then fed into the decoder's input layer (Figure 2). We used PyTorch (Paszke et al., 2019) with CUDA support for building all deep neural network models in this study. Given the architecture of the DNN and its inputs and outputs, we can now examine how the DNN is trained.

### DNN Training

We defined the model's loss function as a weighted combination (Eq. 1) of reconstruction loss captured by mean square error (MSE) loss (Eq. 2) and the Kullback–Leibler (KL) divergence loss (Eq. 3). We set the weighting parameter, w, to 0.1 so that the MSE loss has a higher weight (1–0.1 = 0.9) than the KL divergence loss (0.1). We arrived at this weighting scheme from our preliminary experiments through grid search, wherein we observed that setting the KL divergence’s weight to 0.1 helped in the faster convergence of the model loss and in achieving a much better reconstruction accuracy for the trained model, on both the training dataset and validation dataset.

$$Loss_{model}=\left(1-w\right)*Loss_{MSE}\;+\;\left(w\right)*Loss_{KL}$$ (1)

$$Loss_{MSE}=\frac{1}{n}\sum_{i=1}^{n}{\left(Y_{i}-\;{\hat{Y}}_{i}\right)}^{2}$$ (2)

$$Loss_{KL}=D_{KL}\;\left\{N\left[{\left(\mu_{1},\;\ldots ,\mu_{n}\right)}^{T},\;diag\left(\sigma_{1}^{2},\;\sigma_{n}^{2}\right)\right]||N\left(0,\;I\right)\right\}=\;\frac{1}{2}\sum_{i=1}^{n}\left[\sigma_{i}^{2}+\;\mu_{i}^{2}-\text{ln}\left(\sigma_{i}^{2}\right)-1\right]\;$$ (3)

The KL Divergence upon which Eq. 3 is based represents a special case involving the KL-divergence between a multivariate normal distribution, $N\left[{\left(\mu_{1},\;\ldots ,\;\mu_{n}\right)}^{T},\;diag\left(\sigma_{1}^{2},\;\ldots ,\;\sigma_{n}^{2}\right)\right]$ with means $\mu_{1},\;\ldots ,\;\mu_{n}$ and variances $\sigma_{1}^{2},\;\ldots ,\;\sigma_{n}^{2}$, and a standard normal distribution, N(0, I) .

For the minimization of the loss and, thus, the training of the model, we used the Adam optimizer proposed by (Kingma and Ba, 2014) and with the modifications proposed by (Reddi et al., 2019). We initialized the learning rate to 1e-4, the betas [which are used for computing the running averages of gradient and its square (Paszke et al., 2019)] to 0.9 and 0.999, and the weight decay (which is a form of L2 regularization penalty) to 0.01. We used a multistep learning rate scheduler to gradually reduce the learning rate as the training proceeds through 50 equally distributed epoch milestones. At each of the milestones, the new learning rate is obtained by multiplying the current learning rate by 0.99. We used a batch size of 512 and set out to run 5,000 epochs in the initial training of the model. We adopt early stopping if the model does not improve over 250 consecutive epochs, in which case we would retain the last known best model and stop further training of the model.

### DESP: Deep Enhanced Sampling of Proteins’ Conformation Space Algorithm

**FIGURE 3:**
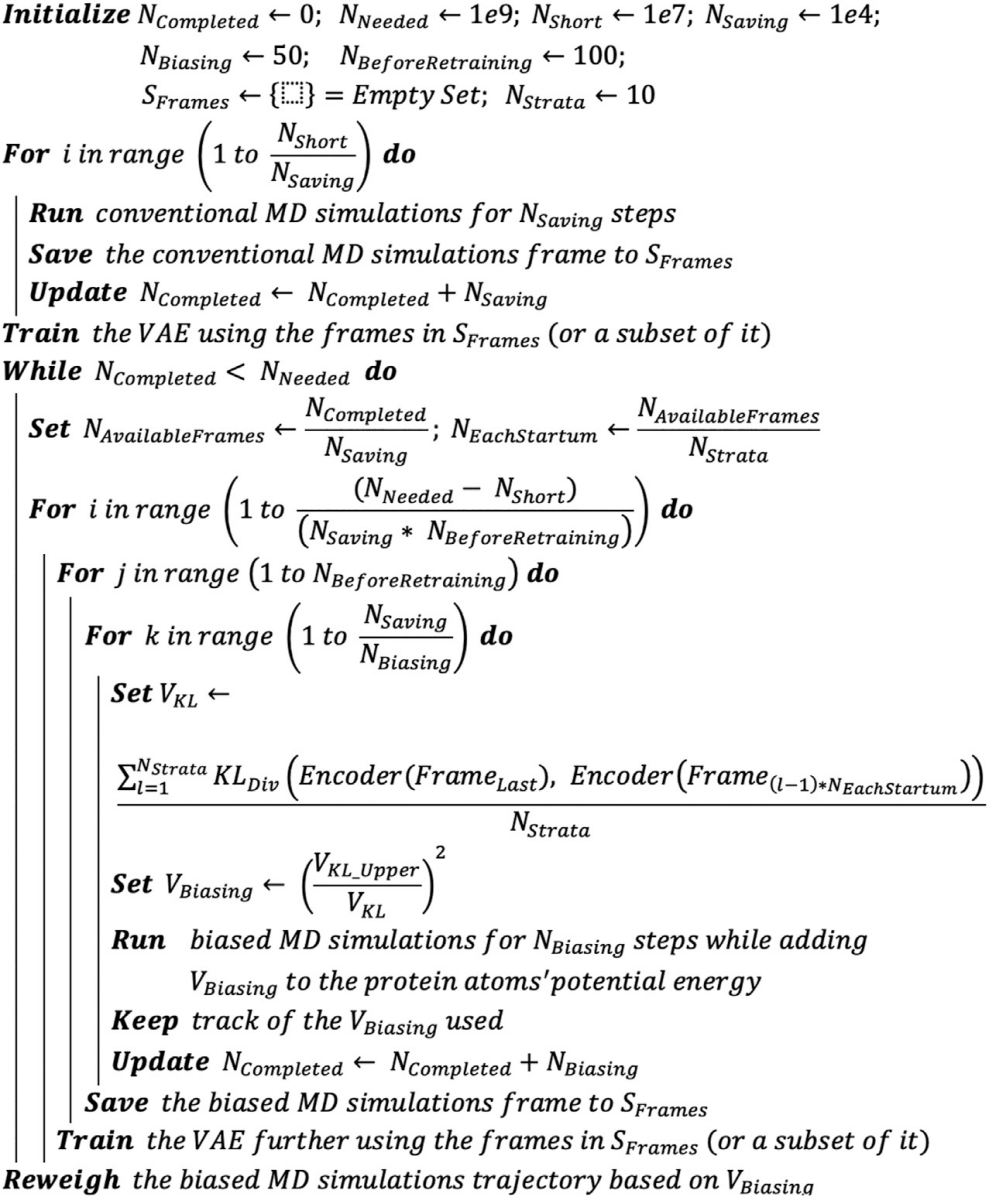
DESP algorithm. The DESP combines DNNs with MD simulations to achieve enhanced sampling of molecules’ conformation spaces.

Having described the individual components of the DESP above, we now present the overall DESP algorithm (Figure 3) that combines DNN with MD simulations to achieve enhanced sampling of the conformation space of macromolecules. It begins with the initialization of the total number of MD simulation steps needed (e.g., N_Needed_ = 1e9), the number of MD simulation steps for the initial short MD run (N_Short_ = 1e7) that will be used for the initial DNN model training, the total number of steps completed (N_Completed_ = 0), the number of steps to run before saving a frame (N_Saving_ = 1e4), and the total number of steps completed before updating the biasing potentials (N_Biasing_ = 50). While N_Completed_ essentially ranges from 0 to N_Needed_ over time, the other variables are relatively as follows:

$$N_{Biasing}\ll \;N_{Saving}\ll \;N_{Short}\ll \;N_{Needed}\;$$ (4)

We use “<<” to signify a difference of one order of magnitude or more. N_Biasing_, N_Saving_, N_Short_, N_Needed_, and N_Completed_ are natural numbers.

We run N_Short_ steps of unbiased MD simulation, saving frames for every N_Saving_ steps. The saved frames are added to a pool of frames (i.e., set S_Frames_): increase N_Completed_ by N_short_ (i.e., N_Completed_ ← N_Completed_ + N_Short_). We use the MD simulation’s frames in S_Frames_ (or its subset, selected randomly) to train the VAE and save the trained VAE (VAE_Trained_). While N_Completed_ is less than N_Needed_, we continue the biased MD simulations coupled with the usage of the VAE_Trained_ and its further training as follows. 1) Calculate the KL divergence (using latent vectors of the VAE_Trained_’s means and variance, based on Eq. 5) between the last frame of the MD simulations and the representative/sampled structures from pool S_Frames_. 2) Run the ongoing MD simulation for N_Biasing_ steps, but now by adding a biasing potential (V_Biasing_, as defined in Eq. 6) that is based on the KL divergence. Keep track of the V_Biasing_. Increase N_Completed_ by N_Biasing_ (i.e., N_Completed_ ← N_Completed_ + N_Biasing_). 3) For every N_Saving_ steps of the MD simulations, add the new frame to pool S_Frames_. And for every N_Saving_ * 100 steps of the MD simulations (which means that additional 100 new frames would have been added to S_Frames_), we use the frames in S_Frames_ (or its subset, selected randomly) to further train the VAE_Trained_. When the N_Completed_ is equal to N_Needed_, we stop the MD simulations and use the trajectory of V_Biasing_ to reweigh the MD simulation trajectory.

The KL Divergence upon which the biasing potential is based involves pairs of multivariate normal distributions of the same dimension and can be represented by Eq. 5, which denotes the KL divergence of $N_{1}\;∼\;N\left(\mu_{1},\;\;{\text{Σ}}_{1}\right)$ from $N_{0}\;∼\;N\left(\mu_{0},\;\;{\text{Σ}}_{0}\right)$.

$$V_{KL}=D_{KL}\left(N_{0}\;||N_{1}\right)=\;\frac{1}{2}\left(\text{tr}\left({\text{Σ}}_{1}^{-1}{\text{Σ}}_{0}\right)+{\left(\mu_{1}-\mu_{0}\right)}^{T}\sum_{1}^{-1}\left(\mu_{1}-\mu_{0}\right)-\text{k}+\text{ln }\left(\frac{det{\text{Σ}}_{1}}{det{\text{Σ}}_{0}}\right)\right)$$ (5)

$$V_{biasing}={\left(V_{KL_{upper}}\;/\;V_{KL}\right)}^{2}$$ (6)

where $V_{KL_{upper}}$, which is set to 1e-5 in this work, is a weak upper bound of V_KL_. $V_{KL_{upper}}$ is a settable parameter but can be left at this default value obtained from our preliminary experiments where this provided optimal enhanced sampling without making the system unstable. This value can be tuned up or down to modulate how aggressive (high $V_{KL_{upper}}$) or conservative (low $V_{KL_{upper}}$) the enhancement of the sampling should be. The obtained V_Biasing_ is added to the potential energy term involving the protein atoms.

At this point, we find it important to further clarify that the use of dihedral angles as input to the VAE in DESP does not mean that dihedral angles are being used directly as the reaction coordinates for biasing the MD simulations. Using all the dihedral angles by themselves would be overwhelming (especially for medium-sized to large-sized molecules) and, more importantly, will not work if used directly even with existing enhanced sampling methods. On the other hand, the VAE learns the compressed representation of the molecular system, and it is the compressed representation (obtainable from the latent space vectors of the VAE, see Figure 2) that is used for achieving the biasing, as presented in the algorithm (see Figure 3). In other words, generally, a bias potential V(R) used in the MD simulation would depend on R, the atomistic coordinate, usually through some collective variables. The same is, in principle, true in the current work, except that the bias potential V(R) used in DESP depends on R’, where R’ is a compressed representation of R that is obtained from the DNN.

### Reweighing of the Probability Distribution

The probability, *p*’ (RC), along a reaction coordinate of interest, RC (r), where r represents the atomic coordinates r_1_^3^, … , r_n_^3^, based on the biased MD simulations can be reweighed using V_Biasing_ to obtain the un-normalized probability distribution, *p* (RC), of the canonical ensemble (Sinko et al., 2013; Miao et al., 2015; Salawu, 2018a) as shown in Eq. 7. And the reweighed free energy change can be obtained from Eq. 8.

$$p\left(RC_{a}\right)=p^{'}\left(RC_{a}\right)*\;\frac{e_{a}^{\beta V_{biasing}}}{\sum_{a=1}^{M}e_{a}^{\beta V_{biasing}}}\;for\;a=1,\;\ldots ,\;M$$ (7)

where $\beta =\;-\;\frac{1}{k_{B}T}$.

$$F\left(RC_{a}\right)=\text{β ln}p\left(RC_{a}\right)$$ (8)

## Results and Discussion

### GB98 is a Small Protein With One α- and Four β-Folds, While A_12_ is a Dodecalanine

**FIGURE 4:**
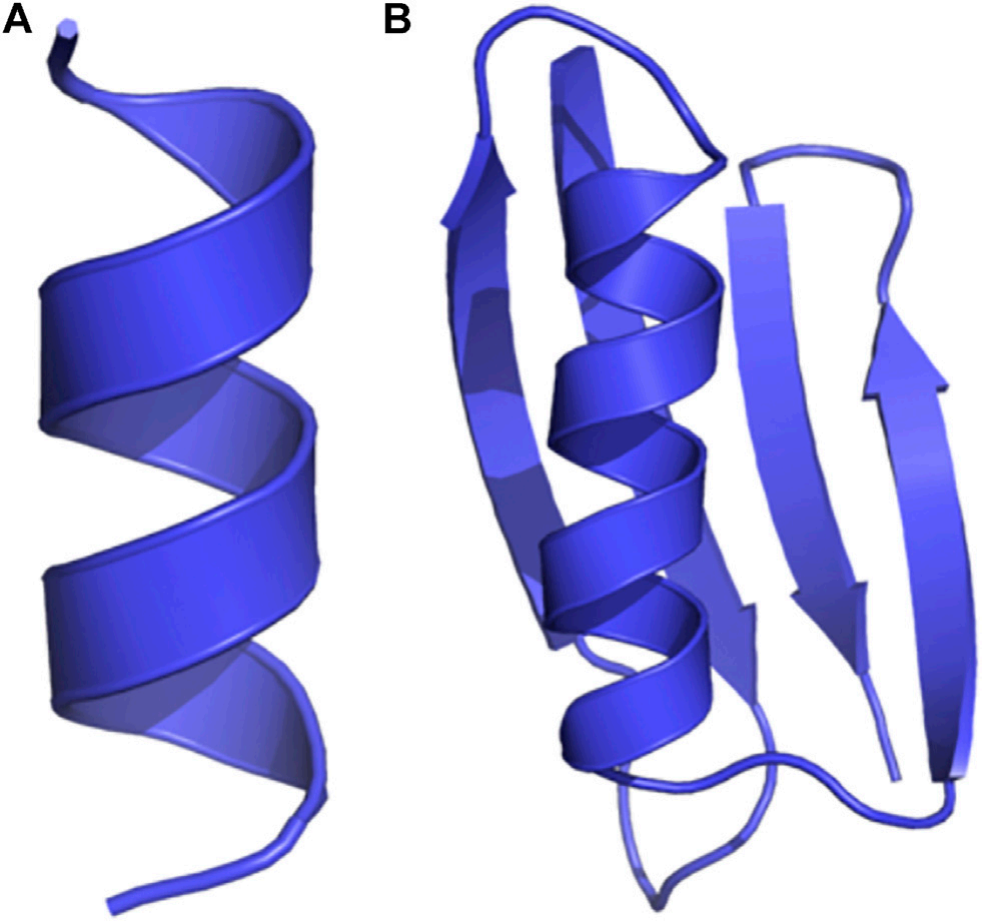
Initial structures of the proteins studied. **(A)** A_12_ is a dodecapeptide with 12 alanine residues, and **(B)** GB98 is a small protein with four beta-sheets and one alpha helix.

We show the initial 3D structure of the studied molecules, A_12_ and GB98, in Figures 4A,B, respectively. A_12_ is a peptide with 12 alanine amino acids, while GB98 is a small protein with four beta-sheets, and one alpha-helix (He et al., 2012; Salawu, 2016). These small-sized and medium-sized molecules helped in illustrating the capabilities of DESP.

### DNN Model Loss During the DESP

**FIGURE 5:**
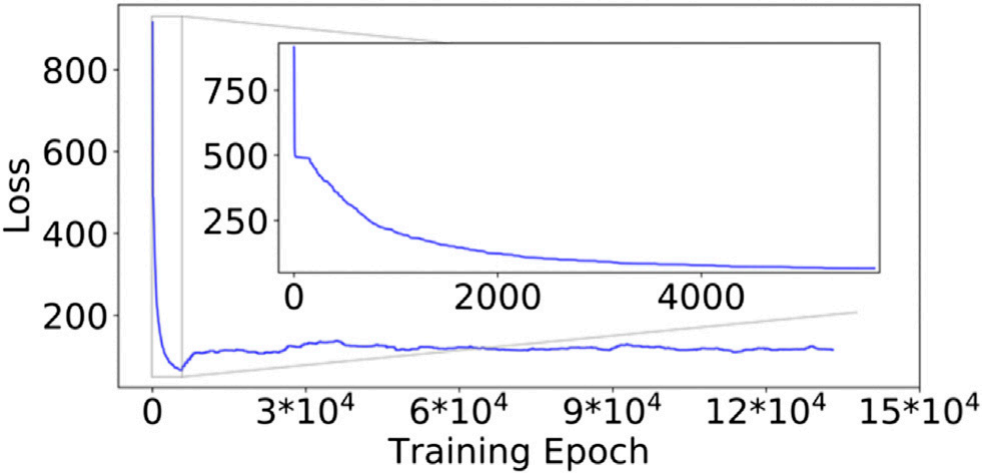
Model loss values during the DESP for the GB98 molecular system. The loss decreased steadily in the first segment of the DESP (see the inset). The model’s loss is slightly higher in the subsequent training of the model because the model was exposed to a more diverse molecular structure. The trajectory of the loss for the A_12_ molecular system is similar in the overall structure/trend to that of the GB98 molecular system and is not shown here for brevity. DESP systematically modifies the molecular system's energy surface.

The initial training of the DESP’s VAE started with a high model total loss (Loss_Model_, Eq. 1) of approximately 915.71 (Figure 5, for the GB98 molecular system), which decreased steadily as the model continued to learn the compressed representation of the molecule under study (inset of Figure 5). The initial model training was stopped when the Loss_Model_ reached 66.65 after 5,664 epochs and would not further decrease for the next 250 epochs. The Loss_Model_ during the subsequent training of the DNN alongside the DNN-biased MD simulations (using the MD simulation’s newly generated molecular structures) is shown in the rest of Figure 5 from epoch 5,664 to the end.

**FIGURE 6:**
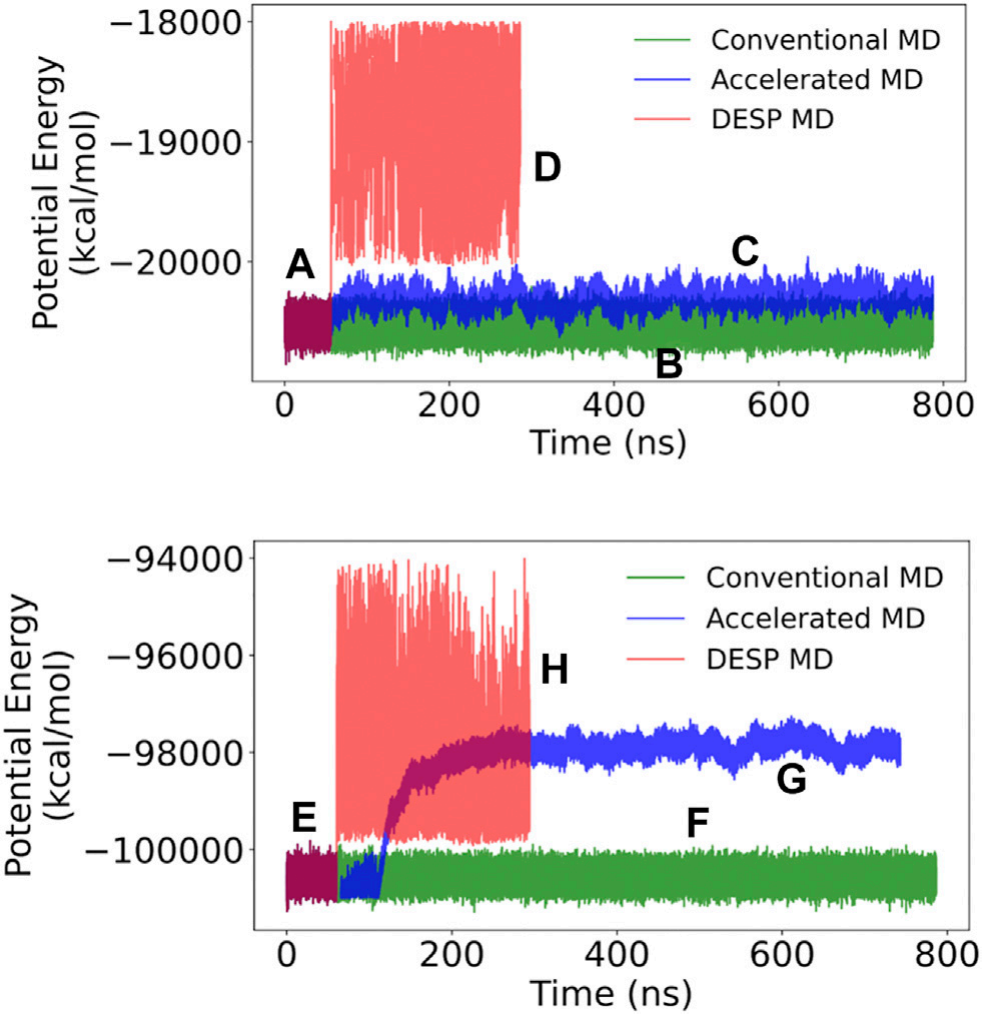
The potential energy for A_12_ (top) and GB98 (bottom) molecular systems. **(A and E)** The initial stages of DESP (as well as the initial stages of accelerated) MD simulations are identical to conventional MD simulations and have identical systems’ potential energies. **(B and F)** The trajectories of the potential energy for the conventional MD simulations are shown in green; **(C and G)** those for the accelerated MD simulations are shown in blue; while **(D and H)** those for the DESP MD simulations are shown in red.

The reader would notice that the Loss_Model_ obtained during the subsequent training of the model alongside the DNN-biased MD simulations is slightly higher than the smallest Loss_Model_ obtained in the initial model training. This is interesting and understandable because the initial training of the DESP’s DNNs was done using only the structures/conformations of the molecule obtained from conventional MD simulations in the first segment of the DESP (Figure 6A), while the subsequent training of the DNN was done using the more structurally diverse conformations of the molecule obtained during the biasing segment of the DESP (Figure 6B).

**FIGURE 7:**
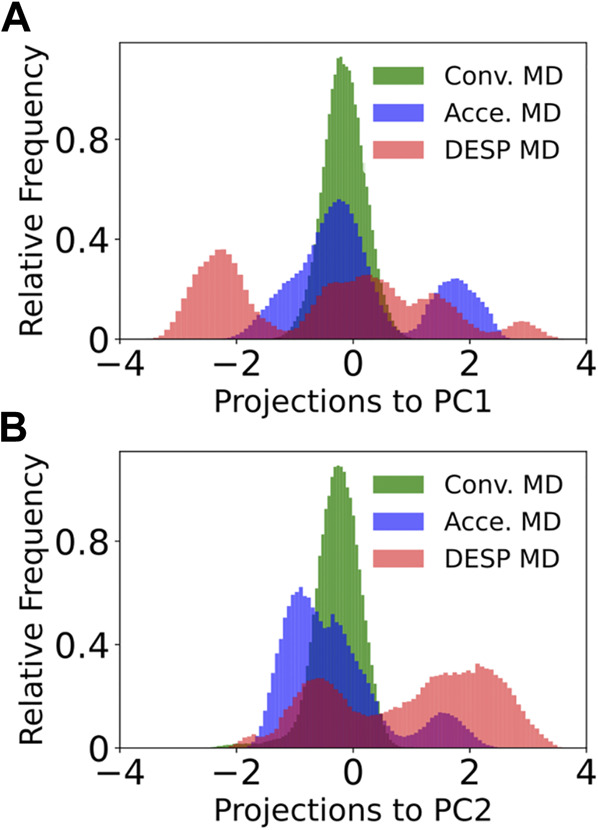
Projections of the trajectory to the first principal component for GB98 (bottom). The projection of each of the frames in the DESP trajectory into the PC1’s space **(A)** and PC2’s space **(B)** are shown for GB98. Similar projections for the A_12_ molecular system do not offer additional information and are now shown here for brevity.

**FIGURE 8:**
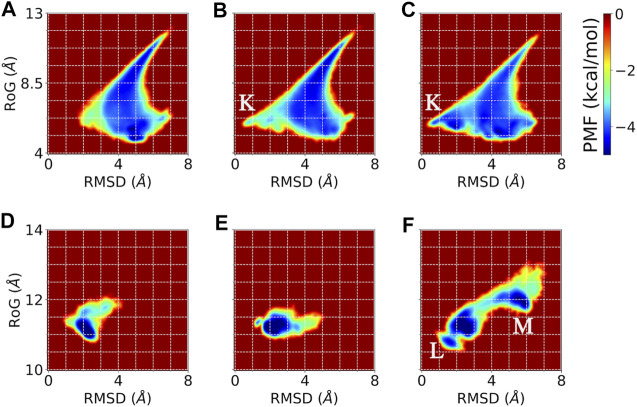
Potentials of mean force (PMF) showing the distribution of the sampled conformations by conventional MD, accelerated MD, and DESP MD simulations of A_12_ (top) and GB98 (bottom) molecular systems. Reweighting has been done wherever necessary. Comparison using PMF based on physically meaningful collective variables, namely, the root mean square deviation from a known experimental/initial structure (RMSD) and the radius of gyration (RoG) are shown for conventional **(A and D)**, accelerated **(B and E)**, and DESP **(C and F)** MD simulations for the A_12_ (top/A, B, C) and the GB98 (bottom/D, E, F) molecular systems. Overall, one would notice that the rightmost panels **(C and D)** show wider and more diverse regions visited by the molecular system, which means that the DESP can explore more conformation spaces than either the conventional **(A and B)** or the accelerated **(B and E)** MD simulations for these collective variables. The regions with stable conformations sampled by both DESP and accelerated MD simulations but not sampled by the conventional MD simulations are marked with “K,” while the regions sampled by DESP alone but not sampled by either the conventional or the accelerated MD simulations are marked with “L” and “M.”

The initial stages of DESP (as well as the initial stage of the accelerated MD) simulations are identical to those of conventional MD simulations, and the molecular systems are observed to have identical systems’ energy surfaces/distributions as conventional MD simulations. Indeed, in the current work, for a given molecular system, after equilibration, the ongoing conventional MD simulation is forked/copied into three: one for continuation as a conventional MD simulation, one for continuation as an accelerated MD simulation, and one for continuation as a DESP MD simulation. At the start of the biasing phase of DESP, we observed that the molecular systems’ energies are modified and the potential energies increase and change based on the conformation being sampled (Figures 6A–D for the A_12_ molecular system, and Figures 6E–H for the GB98 molecular system; not drawn to the same scale for all the MD simulations). The modification of the systems’ potential energies makes it possible for the system to escape possible energy barriers, thereby encouraging the sampling of wider conformation spaces (Figure 7; Figure 8).

### DESP Efficiently Samples a Wider Range of a Molecule’s Conformation Space Than Both Conventional and Accelerated MD Simulations

To compare the conformation spaces sampled by DESP to that sampled by conventional and accelerated MD simulations, we carried out dihedral principal components analysis (dPCA) on the molecule’s dihedral angles (namely, phi, psi, omega, and chi_1_) by making use of both the cosine and the sine of each of the dihedral angles (Mu et al., 2005) and projected each of the sampled structures from the DESP and from both the conventional and accelerated MD simulations into the principal components’ (PC) space. A visualization of the trajectory in the PC space (see Figure 7 for the first two PCs, PC1 and PC2) shows that DESP samples a wider range of the molecule’s conformation spaces than the conventional and the accelerated MD simulations (Figure 7) despite that the DESP is just about one-third as long (i.e., ∼280 ns) as the conventional and the accelerated MD simulations (i.e., ∼800 ns, Figure 6; Figure 7).

It is worthy of note that the distributions shown in Figure 7 are unweighed and cannot be strictly interpreted in the probability sense most especially for the DESP and for the accelerated MD simulations that involve the use of biasing potentials. It is, therefore, important to reweigh any DESP-obtained (or accelerated-MD-obtained) distribution while considering the biasing potentials (Salawu, 2018a; Sinko et al., 2013; Miao et al., 2015). Such reweighting can be achieved through Eqs 7, 8 or as described in previous publications (Sinko et al., 2013; Miao et al., 2015; Salawu, 2018a).

For the potentials of mean force (PMF) obtainable through the reweighting of the trajectories, we use two physically interpretable/physically meaningful reaction coordinates, namely, the molecule’s radius of gyration (RoG) and the molecule’s root mean square deviation (RMSD) from the experimentally solved structure (i.e., the NMR structure in the case of GB98) or the initial structure (i.e., the energy minimized modeled structure in the case of A_12_). The PMF obtained from the reweighed trajectory (Figure 8) further establishes that DESP samples have much wider conformation spaces than the conventional and the accelerated MD simulations. We show the PMF obtained from collective variables (CVs) defined by the combination of the RMSD and the RoG in Figure 8.

From the energy landscape one sees regions with stable conformations that are sampled by DESP but are not sampled by the conventional MD simulations (see Figures 8A–C for the A_12_ molecular system, and Figures 8D–F for the GB98 molecular system). For the sake of illustration, we mark the regions sampled by both DESP and accelerated MD simulations but not sampled by the conventional MD simulations with “K” (Figures 8B,C), and we mark the regions sampled by DESP alone but not sampled by either the conventional MD simulations or the accelerated MD simulations with “L” and “M” (Figure 8F). Overall, one would notice that the rightmost panels (C, D) show wider and more diverse regions visited by the molecular system, which means that the DESP can explore more conformation spaces than either the conventional (A, B) or the accelerated (B, E) MD simulations, to the extent of capturing a few global but moderate unfolding and refolding events. The comparison of the energy landscapes shows that while DESP shows a moderately better sampling of a wider range of conformation spaces than both the conventional and the accelerated MD simulations for a small molecular system (namely, A_12_, Figures 8A–C), the superiority of the sampling efficiency of DESP is more remarkably evident for larger molecules as shown by the medium-sized GB98 molecular system wherein DESP samples much wider regions/conformations spaces than both the conventional and the accelerated MD simulations (Figures 8D–F). This is desirable because it is with the larger molecules that highly efficient conformation space sampling methods, such as DESP, are most needed.

## Conclusion

In this work, 1) It has been shown, with computational experiments and pieces of evidence obtained therefrom, that it is possible to enhance the MD simulation sampling of molecules’ conformation spaces using deep learning techniques (VAE in the current case). 2) It has been shown one of the possible ways with which it could be achieved, namely, by biasing the MD simulations based on the VAE’s latent space vectors. 3) The use of the KL divergence of the DNN-learned latent space vectors of the most recently sampled conformation from the previously sampled conformations made it possible to bias the MD away from visiting already sampled conformations, and thereby encouraging the sampling of previously unsampled states. 4) It should be noted that the ideas in DESP are generalizable and may be used for implementing other forms of biased MD simulations, including targeted and steered MD simulations, and we explore these in our subsequent articles.

## Data Availability

The raw data supporting the conclusions of this article will be made available by the authors, without undue reservation.
